# Edoxaban for 12 vs. 3 months in cancer-associated isolated distal deep vein thrombosis according to different doses: insights from the ONCO DVT study

**DOI:** 10.1093/ehjcvp/pvae028

**Published:** 2024-04-22

**Authors:** Ryuki Chatani, Yugo Yamashita, Takeshi Morimoto, Nao Muraoka, Michihisa Umetsu, Yuji Nishimoto, Takuma Takada, Yoshito Ogihara, Tatsuya Nishikawa, Nobutaka Ikeda, Kazunori Otsui, Daisuke Sueta, Yukari Tsubata, Masaaki Shoji, Ayumi Shikama, Yutaka Hosoi, Yasuhiro Tanabe, Kengo Tsukahara, Naohiko Nakanishi, Kitae Kim, Satoshi Ikeda, Kazunori Mushiake, Kazushige Kadota, Koh Ono, Takeshi Kimura

**Affiliations:** Department of Cardiovascular Medicine, Kurashiki Central Hospital, Kurashiki 7100052, Japan; Department of Cardiovascular Medicine, Graduate School of Medicine, Kyoto University, 54 Shogoin Kawahara-cho, Sakyo-ku, 6068507 Kyoto, Japan; Department of Clinical Epidemiology, Hyogo Medical University, Nishinomiya 6638501, Japan; Division of Cardiology, Shizuoka Cancer Center, Shizuoka 4118777, Japan; Division of Vascular Surgery, Department of Surgery, Tohoku University Hospital, Sendai 9808574, Japan; Division of Cardiology, Osaka General Medical Center, Osaka 5588558, Japan; Department of Cardiology, Tokyo Women's Medical University, Tokyo 1628666, Japan; Department of Cardiology and Nephrology, Mie University Graduate School of Medicine, Tsu 5148507, Japan; Department of Onco-Cardiology, Osaka International Cancer Institute, Osaka 5418567, Japan; Division of Cardiovascular Medicine, Toho University Ohashi Medical Center, Tokyo 1538515, Japan; Department of General Internal Medicine, Kobe University Graduate School of Medicine, Kobe 6500017, Japan; Department of Cardiovascular Medicine, Graduate School of Medical Sciences, Kumamoto University, Kumamoto 8608556, Japan; Department of Internal Medicine, Division of Medical Oncology and Respiratory Medicine, Shimane University Faculty of Medicine, Izumo 6938501, Japan; Department of Cardiovascular Medicine, National Cancer Center Hospital, Tokyo 1040045, Japan; Department of Obstetrics and Gynecology, Faculty of Medicine, University of Tsukuba, Tsukuba 3058576, Japan; Department of Cardiovascular Surgery, Kyorin University Faculty of Medicine, Tokyo 1818611, Japan; Department of Cardiology, St Marianna University School of Medicine, Kawasaki 2168511, Japan; Division of Cardiology, Fujisawa City Hospital, Fujisawa 2518550, Japan; Department of Cardiovascular Medicine, Graduate School of Medical Science, Kyoto Prefectural University of Medicine, Kyoto 6028566, Japan; Department of Cardiovascular Medicine, Kobe City Medical Center General Hospital, Kobe 6500047, Japan; Department of Cardiovascular Medicine, Nagasaki University Graduate School of Biomedical Sciences, Nagasaki 8528501, Japan; Department of Cardiovascular Medicine, Kurashiki Central Hospital, Kurashiki 7100052, Japan; Department of Cardiovascular Medicine, Kurashiki Central Hospital, Kurashiki 7100052, Japan; Department of Cardiovascular Medicine, Graduate School of Medicine, Kyoto University, 54 Shogoin Kawahara-cho, Sakyo-ku, 6068507 Kyoto, Japan; Department of Cardiology, Hirakata Kohsai Hospital, Hirakata 5730153, Japan

**Keywords:** Anticoagulant regimen, Cancer, Cardio-oncology, Deep vein thrombosis, Edoxaban

## Abstract

**Background:**

The ONCO DVT study revealed the superiority of 12-month relative to 3-month edoxaban treatment for cancer-associated isolated distal deep vein thrombosis (DVT) regarding the thrombotic risk.

**Methods and Results:**

In this pre-specified subgroup analysis of the ONCO DVT study, we stratified the patients into those with a standard edoxaban dose (60 mg/day; *N* = 151) and those with a reduced edoxaban dose (30 mg/day; *N* = 450) and evaluated the clinical outcomes for the 12- and 3-month treatments. The cumulative 12-month incidence of symptomatic recurrent venous thromboembolism was lower in the 12-month than 3-month group for both the 60 mg (1.3% vs. 11.6%, *P* = 0.02; odds ratio [OR], 0.12; 95% confidence interval [CI], 0.01–0.97) and 30 mg (1.1% vs. 7.6%, *P* = 0.002; OR, 0.14; 95% CI, 0.03–0.60) edoxaban subgroups, which was consistent across the edoxaban doses without a significant interaction (*P* = 0.90). The 12-month cumulative incidence of major bleeding was higher in the 12-month group than in the 3-month group for the 60 mg edoxaban subgroup (14.3% vs. 4.4%, *P* = 0.046; OR, 3.61; 95% CI, 0.97–13.52), whereas it did not significantly differ between the two groups for the 30 mg edoxaban subgroup (8.7% vs. 8.6%, *P* = 0.89; OR, 0.97; 95% CI, 0.49–1.91), signalling there was a potential interaction (*P* = 0.07).

**Conclusions:**

A 12-month edoxaban regimen for cancer-associated isolated distal DVT was consistently superior to a 3-month regimen, across the edoxaban doses for the thrombotic risk. However, caution was suggested for the standard dose of edoxaban due to the potential for an increased risk of bleeding with prolonged anticoagulation therapy.

**Trial registration number:**

NCT03895502 (ONCO DVT Trial): https://classic.clinicaltrials.gov/ct2/show/NCT03895502

## Abbreviations

DOAC: direct oral anticoagulant
DVT: deep vein thrombosis
PE: pulmonary embolism
VTE: venous thromboembolism

## Introduction

Cancer-associated isolated distal deep vein thrombosis (DVT) is reported to account for 11% of all cancer-associated thromboses and negatively impacts the prognosis in cancer patients, with a similar effect as that of proximal DVT and pulmonary embolism (PE).^1^ A previous study reported that the incidence rate of recurrent venous thromboembolism (VTE) in patients with cancer-associated isolated distal DVT is 5.65 per 100 person-years.^2^ Another study reported that cancer patients with isolated distal DVT have an approximately 3-fold higher risk for recurrent VTE than non-cancer patients.^3^ Therefore, patients with cancer-associated isolated distal DVT have been considered to be at a relatively high risk of recurrent VTE.^4–7^ A recent randomized clinical trial, the ONCO DVT study, showed the potential benefit of an extended anticoagulation therapy with edoxaban for patients with cancer-associated isolated distal DVT in terms of the thrombotic risk.^8^ However, there has been some concern about the increased risk of bleeding with prolonged anticoagulation therapy in patients with cancer-associated VTE. Thus, an individual risk assessment and treatment strategy could be important for the optimal management strategies in these patients.

The doses of the direct oral anticoagulants (DOACs) could be a clinically relevant issue for extended anticoagulation therapy in patients with VTE,^9,10^ which might also be important in patients with cancer-associated VTE. However, there has been limited data on this issue in patients with cancer-associated VTE. Furthermore, the issue might be especially important in patients with cancer-associated isolated distal DVT considering the potentially lower risk of thrombotic events as compared with those with PE and proximal DVT.^11,12^ Therefore, we conducted this pre-specified subgroup analysis according to the different doses of edoxaban to compare the efficacy and safety between a 12-month and 3- edoxaban treatment in patients with cancer-associated isolated distal DVT using the ONCO DVT study database.

## Methods

### Trial design and oversight

The ONCO DVT study (NCT03895502) was an investigator-initiated, multicentre, open-label, adjudicator-blinded, superiority, randomized clinical trial conducted at 60 institutions in Japan that was designed to compare 12- and 3-month edoxaban treatment regimens in patients with cancer and isolated distal DVT (*Appendix S1*).^8^ The full details of the ONCO DVT study were described previously.^8^ Just briefly, patients with active cancer who were newly diagnosed with isolated distal DVT using ultrasonography were randomly assigned, in a 1-to-1 ratio, either to the 12- or 3-month edoxaban treatment group. The trial was conducted in accordance with the principles of the Declaration of Helsinki and was approved by the Kyoto University Institutional Review Board and the institutional review boards of all participating institutions (*Appendix S2*).

### Study population

**Figure 1:**
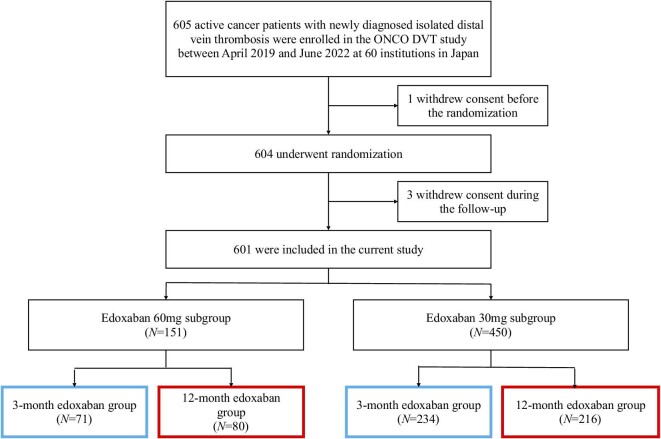
Study flow chart. DVT, deep vein thrombosis.

From April 2019 to June 2022, 604 patients were randomized. After excluding 3 patients who withdrew consent during follow-up, 601 patients were included in the current study (*Figure 1*). Based on the initial doses of edoxaban, the study population was divided into the standard dose (60 mg per day) and reduced dose (30 mg per day) subgroups.

Edoxaban was administered after an appropriate initial treatment after the diagnosis, in accordance with the policies at each institution. No restrictions were set for the policies, providing the treatment did not contravene the exclusion criteria. In the study protocol, edoxaban was recommended to be administered at a reduced dose of 30 mg once daily in patients with a creatinine clearance of 30–50 mL per minute, a body weight of ≤60 kg, or receiving concomitant treatment with potent P-glycoprotein inhibitors.

### Patient characteristics and definitions

Active cancer was defined as cancer satisfying one of the following criteria: newly diagnosed within 6 months of randomization; cancer treatment (such as surgery, chemotherapy, or radiotherapy) performed within 6 months of randomization; currently receiving cancer treatment (such as surgery, chemotherapy, or radiotherapy); having a recurrence, local invasion, or distant metastases; or patients with a haematopoietic malignancy who had not achieved complete remission.^1^ Definitions of the other baseline characteristics are provided in *Appendix S3*.

### Clinical outcomes and definitions

The primary and major secondary endpoints of the current study were identical to those used in the primary report.^1^ The primary endpoint was a composite of symptomatic recurrent VTE and VTE-related death at 12 months. Symptomatic recurrent VTE was defined as new or recently worsening PE or DVT symptoms, new thrombi found on imaging tests, or worsening thrombi compared with the most recent image. Symptomatic VTE recurrence was not determined solely based on the appearance or worsening of thrombus images without new or worsening symptoms. Similarly, if a patient had a thrombus in an index vein with new symptoms, it was not considered as symptomatic recurrent VTE unless a thrombus extension was present. VTE-related death was diagnosed at autopsy, following a clinically severe PE or death unexplained by something other than a PE. The major secondary endpoint was major bleeding events at 12 months. Major bleeding was defined according to the International Society on Thrombosis and Haemostasis (ISTH) criteria, which was comprised of fatal bleeding, symptomatic bleeding in a critical area or organ, and bleeding reducing the haemoglobin levels by ≥2 g/dL or requiring transfusions of ≥2 units of whole blood or red cells.^13^

Other secondary endpoints were symptomatic recurrent VTE, VTE-related death, asymptomatic recurrent VTE events, all clinically relevant bleeding events, and all-cause death. An asymptomatic VTE event was based on new or worsening thrombus images in any imaging tests during follow-up without any symptoms, which was defined as the appearance of new or worsening thrombus images in the pulmonary arteries and deep veins on imaging tests (ultrasonography of the lower limb vein system, computed tomography examinations, pulmonary perfusion scintigraphy, pulmonary angiography, and venography) that did not match the definition of a symptomatic VTE recurrence and was not associated with new or worsening symptoms. All clinically relevant bleeding events included major and non-major bleeding events. Clinically relevant non-major bleeding was defined as clinically overt bleeding (including bleeds detected only using imaging) not meeting the criteria for major bleeding, yet led to one or more of the following: physician-guided medical intervention, hospital admission or further treatment for bleeding, or in-person medical examination by a physician.

Persistent edoxaban discontinuation was defined as the discontinuation of edoxaban according to the study protocol or lasting >14 days for any reason. Death was adjudicated as caused by VTE, bleeding, cancer, cardiovascular disease, or other causes. The members of an independent clinical outcomes committee who were unaware of the study group assignments adjudicated all the suspected outcome events and causes of death, as well as the severity of the major bleeding events, with the use of pre-specified criteria.^14^ The full details of the definitions of the endpoints are described in *Appendix S4*.

### Statistical analysis

Categorical variables were presented as numbers and percentages, and continuous variables were presented as the mean and standard deviation or median and interquartile range (IQR) based on their distributions. Categorical variables were compared using the χ^2^ test or Fisher's exact test, and continuous variables were compared using the Student's *t*-test or Wilcoxon rank-sum test according to their distributions. The cumulative incidence was estimated using the Kaplan–Meier method, and the differences between the 12- and 3-month edoxaban treatment groups were compared using the log-rank test. As a sensitivity analysis, we also evaluated major bleeding and all clinically relevant bleeding events on edoxaban treatment, which was defined as bleeding events before persistent discontinuation of edoxaban. We calculated the odds ratios (OR) with the corresponding 95% confidence intervals (CI) using logistic regression models, and the differences in the effects of the 12- and 3-month edoxaban treatment regimens were evaluated according to the subgroups of the doses of edoxaban using interaction terms in the models. We also performed a per-protocol and as-treated analysis to avoid any bias of an open-label assigned group for the sensitivity analyses (*Appendix S5*). In addition, to investigate the influence of age, body weight, and creatinine clearance on the clinical endpoints in the reduced-dose edoxaban subgroup, we conducted a further subgroup analysis. The reported *P*-values were two-tailed, and statistical significance was set at *P* < 0.05. The SPSS (IBM Japan, Tokyo) software package (ver. 23) or JMP version 15.2.0 software (SAS Institute Inc, Cary, NC, USA) was used for all analyses.

## Results

### Patient enrolment and characteristics

Among 601 patients included in the current study, 151 (25.1%) and 450 (74.9%) received a standard dose of 60 mg and a reduced dose of 30 mg, respectively (*Figure 1*). The patients in the standard-dose subgroup were younger (67.6 vs. 71.9 years, *P* < 0.001), more often men (53.6% vs. 19.1%, *P* < 0.001), had a higher mean body weight (70.3 vs. 50.6 kg, *P* < 0.001), and had a higher creatinine clearance (86.4 vs. 60.0 mL/min, *P* < 0.001) (*Table 1*). The clinical characteristics of the patients at baseline were well balanced between the 12- and 3-month edoxaban groups in both subgroups (*Table S1*). The details of the cancer types are presented in *Table S2*.

**Table 1:** Baseline clinical characteristics

	Standard dose of edoxaban (60 mg/day) (*N* = 151)	Reduced dose of edoxaban (30 mg/day) (*N* = 450)	*P*-value
**Baseline characteristics**			
Age, years	67.6 ± 9.8	71.9 ± 9.7	<0.001
Age ≥75 years, *n* (%)	43 (29)	202 (45)	<0.001
Men, *n* (%)	81 (54)	86 (19)	<0.001
Body weight, kg	70.3 ± 8.8	50.6 ± 8.1	<0.001
Body weight ≤60 kg, *n* (%)	4 (2.6)	422 (94)	<0.001
Body mass index, kg/m^2^	26.7 ± 4.0	21.1 ± 3.0	<0.001
Symptoms at baseline, *n* (%)	27 (18)	95 (21)	0.42
Site of thrombosis, *n* (%)			
Bilateral, *n* (%)	51 (34)	172 (38)	0.051
Right side, *n* (%)	50 (33)	104 (23)	
Left side, *n* (%)	50 (33)	174 (39)	
**Cancer status**			
Newly diagnosed with cancer within 6 mo, *n* (%)	103 (68)	286 (64)	0.33
Chemotherapy performed within 6 mo, *n* (%)	68 (45)	215 (48)	0.57
Radiotherapy performed within 6 mo, *n* (%)	8 (5.3)	44 (9.8)	0.10
Scheduled to be operated within 6 mo, *n* (%)	61 (40)	195 (43)	0.57
Local invasion, *n* (%)	26 (17)	94 (21)	0.35
Recurrent cancer, *n* (%)	11 (7.3)	54 (12)	0.13
Metastatic disease, *n* (%)	34 (23)	113 (25)	0.59
ECOG performance status, *n* (%)			
0	88 (58)	223 (50)	0.11
1	43 (29)	138 (31)	
≥2	20 (13)	89 (20)	
**Comorbidities**			
Hypertension, *n* (%)	77 (51)	186 (41)	0.046
Diabetes, *n* (%)	46 (31)	55 (12)	<0.001
Heart failure, *n* (%)	4 (2.6)	6 (1.3)	0.28
History of stroke, *n* (%)	8 (5.3)	19 (4.2)	0.65
History of VTE, *n* (%)	10 (6.6)	23 (5.1)	0.54
History of major bleeding, *n* (%)	7 (4.6)	16 (3.6)	0.62
Transient risk factors for VTE, *n* (%)	37 (25)	114 (25)	0.91
Recent surgery within 2 months, *n* (%)	22 (15)	63 (14)	0.89
**Laboratory tests at diagnosis**			
Creatinine clearance, mL/min	86.4 (69.8–106.3)	60.0 (47.3–75.3)	<0.001
Creatinine clearance ≤50 mL/min, *n* (%)	3 (2.0)	128 (28)	<0.001
Anaemia, *n* (%)	87 (58)	315 (70)	0.007
Platelet count <100 000 per μL, *n* (%)	9 (6.0)	22 (4.9)	0.67
D-dimer, μg/mL	4.5 (2.1–9.9)	5.1 (2.3–11.4)	0.45
**Concomitant medication**			
Antiplatelet, *n* (%)	10 (6.6)	38 (8.4)	0.60
Steroid, *n* (%)	13 (8.6)	64 (14)	0.09
Statins, *n* (%)	35 (23)	99 (22)	0.82
**Edoxaban dose criteria**			
Body weight >60 kg, and creatinine clearance >50 mL/min, *n* (%)	144 (95)	18 (4.0)	<0.001
Body weight ≤60 kg, or creatinine clearance ≤50 mL/min, *n* (%)	7 (4.6)	432 (96)	<0.001
Potent P-glycoprotein inhibitors for dose reduction of edoxaban, *n* (%)	—	3 (0.7)	—

Values are expressed as the mean ± standard deviation, median (interquartile range), or number with percentage. Categorical variables were compared using the χ^2^ test. Continuous variables were compared using the Student's *t*-test or Wilcoxon's rank-sum test based on their distributions.

In the study protocol, edoxaban was recommended to be administered at a reduced dose of 30 mg once daily in patients with a creatinine clearance of 30–50 mL per minute, a body weight of ≤60 kg, or receiving concomitant treatment with potent P-glycoprotein inhibitors. The values of the D-dimer were missing in 34 patients.

Abbreviations: ECOG, Eastern Cooperative Oncology Group; VTE, venous thromboembolism.

### Edoxaban treatment

Among the 60 mg edoxaban subgroup, the cumulative 120-day incidence of persistent edoxaban discontinuation was 22.5% and 84.2% in the 12- and 3-month edoxaban groups, respectively (*Figure S1A*). In the 30 mg edoxaban subgroup, the cumulative 120-day incidence of persistent edoxaban discontinuation was 20.9% and 86.9% in the 12- and 3-month edoxaban groups, respectively (*Figure S1B*). The details of the reasons for a persistent edoxaban discontinuation are presented in *Table S3*.

### Primary endpoint

**Figure 2:**
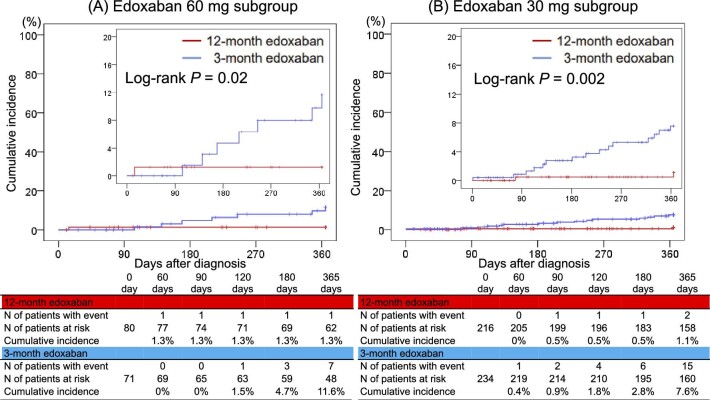
Kaplan–Meier curves for the primary endpoint comparing the 12- and 3-month edoxaban treatment groups in the subgroups stratified by the doses of edoxaban: (A) edoxaban 60 mg subgroup and (B) edoxaban 30 mg subgroup. The primary endpoint was a composite of symptomatic recurrent venous thromboembolism or venous thromboembolism-related death.

The cumulative incidence of the primary endpoint was lower in the 12-month edoxaban group than in the 3-month edoxaban group in the 60 mg edoxaban subgroup (1.3% vs. 11.6%, log-rank *P* = 0.02) (*Figure 2**A*) and the 30 mg edoxaban subgroup (1.1% vs. 7.6%, log-rank *P* = 0.002) (*Figure 2**B*). The 12-month edoxaban group had a lower risk of the primary endpoint than the 3-month edoxaban group in both the 60 mg edoxaban subgroup (OR, 0.12; 95% CI, 0.01–0.97) and the 30 mg edoxaban subgroup (OR, 0.14; 95% CI, 0.03–0.60), and there was no significant interaction (*P* = 0.90) (*Table 2*). The results of the per-protocol and as-treated analyses were generally consistent with the results of the primary analysis (*Tables S4* and *S5*, and *Figures S2*–*S5*).

**Table 2:** Clinical outcomes at 12 months by comparing the 12- and 3-month edoxaban groups with the standard dose (60 mg/day) and reduced dose (30 mg/day) edoxaban subgroups^a^

	Number of patients with event (cumulative 1-year incidence)			
	12-month edoxaban (*N* = 296)	3-month edoxaban (*N* = 305)	Odds ratio (95% CI)	*P*-interaction
**Primary endpoint**				
60 mg/day	1/80 (1.3%)	7/71 (11.6%)	0.12 (0.01–0.97)	
				0.90
30 mg/day	2/216 (1.1%)	15/234 (7.6%)	0.14 (0.03–0.60)	
**Major secondary endpoint** ^b^				
60 mg/day	11/80 (14.3%)	3/71 (4.4%)	3.61 (0.97–13.52)	
				0.07
30 mg/day	17/216 (8.7%)	19/234 (8.6%)	0.97 (0.49–1.91)	
**Other secondary endpoints**				
*Symptomatic VTE recurrence events*				
60 mg/day	1/80 (1.3%)	7/71 (11.6%)	0.12 (0.01–0.97)	
				0.90
30 mg/day	2/216 (1.1%)	15/234 (7.6%)	0.14 (0.03–0.60)	
*VTE-related deaths* ^c^				
60 mg/day	0/80 (0%)	0/71 (0%)	—	
				—
30 mg/day	0/216 (0%)	0/234 (0%)	—	
*Asymptomatic recurrent VTE* ^d^				
60 mg/day	11/80 (15.5%)	10/71 (16.9%)	0.97 (0.39–2.45)	
				0.06
30 mg/day	12/216 (6.1%)	36/234 (18.5%)	0.32 (0.16–0.64)	
*All clinically relevant bleeding events* ^e^				
60 mg/day	22/80 (28.0%)	6/71 (8.7%)	4.11 (1.56–10.84)	
				0.006
30 mg/day	31/216 (15.7%)	35/234 (15.9%)	0.95 (0.57–1.61)	
*Deaths from all causes*				
60 mg/day	13/80 (16.7%)	16/71 (22.8%)	0.78 (0.34–1.78)	
				0.49
30 mg/day	53/216 (24.8%)	61/234 (26.2%)	0.92 (0.60–1.41)	

^a^The analyses included all the patients who had undergone randomization after excluding patients who withdrew consent. For patients who did not experience an event, the time to the first event was to be censored at day 365, or the last day the patient had a complete assessment for the study outcomes, whichever comes first. We calculated the odds ratios, computed using the logistic regression model along with the corresponding 95% confidence intervals for all clinical endpoints, which have not been adjusted for multiple comparisons.

b Major bleeding events were classified according to the criteria of the International Society on Thrombosis and Hemostasis.

c Death due to VTE was diagnosed prior to death or at autopsy, or death was unexplained by other than a pulmonary embolism.

d Asymptomatic recurrent VTE was defined as the appearance of new or worsening thrombus images in the pulmonary arteries and deep veins on imaging tests (ultrasonography of lower limb vein system, computed tomography examination, pulmonary perfusion scintigraphy, pulmonary angiography, or venography) that did not match the definition of a symptomatic VTE recurrence and was not associated with new or worsening symptoms.

e For patients who had more than one event, only the first was counted.

Abbreviation: VTE, venous thromboembolism.

### Major secondary endpoint

**Figure 3:**
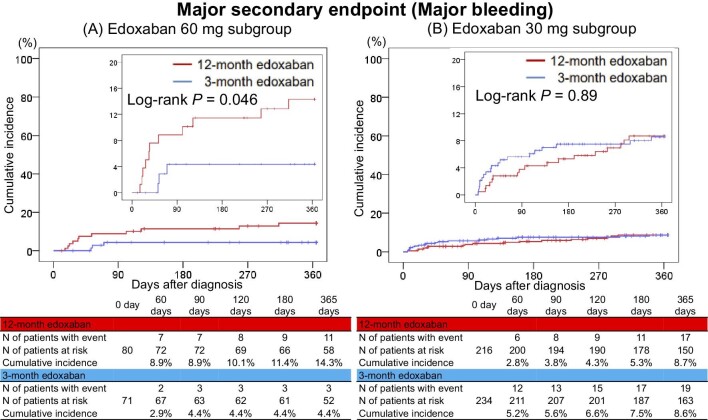
Kaplan–Meier curves for the major secondary endpoint comparing the 12- and 3-month edoxaban treatment groups in the subgroups stratified by the doses of edoxaban: (A) edoxaban 60 mg subgroup and (B) edoxaban 30 mg subgroup. The major secondary endpoint was major bleeding as defined according to the International Society on Thrombosis and Haemostasis criteria, and was comprised of fatal bleeding, symptomatic bleeding in a critical area or organ, and bleeding causing a reduction in the haemoglobin levels of ≥2 g/dL or leading to a transfusion of ≥2 units of whole blood or red cells.

The cumulative incidence of the major secondary endpoint (major bleeding) was higher in the 12-month edoxaban group than in the 3-month edoxaban group in the 60 mg edoxaban subgroup (14.3% vs. 4.4%, log-rank *P* = 0.046) (*Figure 3**A*). However, there was no significant difference in the cumulative incidence of the major secondary endpoint between the 12- and 3-month edoxaban groups in the 30 mg edoxaban subgroup (8.7% vs. 8.6%, log-rank *P* = 0.89) (*Figure 3**B*). The 12-month edoxaban group had a numerically higher risk of the major secondary endpoint than the 3-month edoxaban group among the 60 mg edoxaban subgroup (OR, 3.61; 95% CI, 0.97–13.52); however, there was no significant difference among the 30 mg edoxaban subgroup (OR, 0.97; 95% CI, 0.49–1.91), and there was a potential interaction between the subgroups and the effect of the 12-month edoxaban treatment relative to that of the 3-month edoxaban treatment (*P* = 0.07) (*Table 2*).

### Other secondary endpoints and sensitivity analysis for bleeding events

Other secondary endpoints and the full details of the clinical endpoints are described in *Table 2*, *Figures S6*–*S8*, and *Table S6*. The cumulative incidence of all clinically relevant bleeding events was higher in the 12-month edoxaban group than in the 3-month edoxaban group in the 60 mg edoxaban subgroup (28.0% vs. 8.7%, log-rank *P* = 0.003) (*Figure S7A*). However, there was no significant difference in the cumulative incidence of all clinically relevant bleeding events between the 12- and 3-month edoxaban groups in the 30 mg edoxaban subgroup (15.7% vs. 15.9%, log-rank *P* = 0.80) (*Figure S7B*). There was a significant interaction (*P* = 0.006) (*Table 2*). The sensitivity analysis evaluating major bleeding and all clinically relevant bleeding events on edoxaban treatment is described in *Figures S9* and *S10*, which was fully consistent with the main analysis.

### Further subgroup analysis

The further subgroup analysis for the primary endpoint and major secondary endpoint according to the age, body weight, and creatinine clearance in the reduced-dose edoxaban subgroup is shown in *Table S7*. Even patients with old age, low body weight, and low creatinine clearance had a consistent trend of the results as the main analysis.

## Discussion

The current study could provide some useful insight for the doses of edoxaban. The main findings were as follows: 1) The longer regimen was superior in reducing the risk of symptomatic recurrent VTE across both the standard (60 mg/day) and reduced (30 mg/day) edoxaban doses. 2) The longer regimen had a higher risk of bleeding among the patients receiving the standard dose, but not among those receiving the reduced dose.

For the treatment of acute VTE, the initial adequate anticoagulation therapy could be an important issue because the risk of thrombotic events could be especially high in the acute phase.^15^ However, intensive anticoagulation therapy with an extended duration could provoke concerns for an increased risk of bleeding in the long-term. Edoxaban is administered at a fixed standard dose of 60 mg once daily. If patients have renal dysfunction, a low body weight, or concomitant medications, edoxaban is administered at a reduced dose of 30 mg once daily, which could have a potential benefit of a lower risk of bleeding. This issue might be especially relevant for relatively low-risk VTE, such as isolated distal DVT as well as specific populations including cancer patients, who could often have a high bleeding risk profile.

### Doses of edoxaban and recurrent VTE in cancer patients

In the Hokusai-VTE Cancer study^16^ evaluating PE and proximal DVT in cancer patients, the incidence of recurrent VTE was 7.3% per year in the standard-dose edoxaban subgroup, but was 9.8% per year in the reduced-dose edoxaban subgroup. The edoxaban group showed that the incidence of recurrent VTE was comparable to that for low-molecular-weight heparin irrespective of the doses of edoxaban. The current study evaluating isolated distal DVT showed a lower incidence of symptomatic recurrent VTE with the extended anticoagulation therapy irrespective of the doses of edoxaban, and no fatal VTE events occurred. That could be partly due to the difference in the isolated distal DVT and clinical endpoint, including asymptomatic recurrent VTE events. A previous study reported that patients with isolated distal DVT had less VTE deterioration than those patients with proximal DVT despite a more frequent discontinuation of a prolonged anticoagulant therapy; however, the proportion of cancer patients was relatively small.^11^ A recent study demonstrated that patients with cancer-associated distal DVT less often develop a fatal PE than those with cancer-associated proximal DVT; however, those with cancer-associated distal DVT could have a comparable risk of recurrent VTE as those with cancer-associated proximal DVT.^3^ Considering the potentially lower impact on PE-related mortality of isolated distal DVT, a low dose of anticoagulation therapy could be a reasonable option for the prevention of thrombotic events in these patients.

### Doses of edoxaban and bleeding in cancer patients

In the Hokusai-VTE Cancer study, the incidence of major bleeding was 6.0% per year in the standard-dose edoxaban subgroup, whereas it was 9.8% per year in the reduced-dose edoxaban subgroup.^16^ The current study showed that the 12-month incidence of major bleeding was 14.3% in the standard-dose subgroup and 8.7% in the reduced-dose subgroup in the patients receiving extended edoxaban. A potential heterogeneity in the treatment effect between the standard dose and reduced doses of edoxaban on major bleeding was suggested (interaction *P* = 0.07). The incidence of major bleeding was markedly higher among patients with a standard dose of edoxaban in the current study than in those in the Hokusai-VTE Cancer study. That could be partly due to the difference in the study populations including the cancer status and the races. A previous study reported that patients with active cancer had a markedly high risk of major bleeding.^17^ The current study included only active cancer patients, whereas the Hokusai-VTE Cancer study included a minority of non-active cancer patients, which might have had some influence on the bleeding risk. Another previous study reported that Asian patients had a higher risk of bleeding than non-Asian patients with anticoagulation therapy.^18^ A reduced dose of edoxaban could have the benefit of lowering the risk of bleeding in these patients. In addition, the dose reduction of edoxaban was intended for use in patients with a high risk of bleeding, such as those with renal dysfunction and a low body weight.

On the other hand, various factors have been identified as adaptation criteria, which are reported to be independently associated with an increased bleeding risk.^19^ Notably, the pharmacological rationale for the dose reduction could be reflected by issues of bioaccumulation of edoxaban rather than a reduction in the bleeding risk at the expense of the efficacy. Considering the balance between the risk of thrombotic events and bleeding events in cancer-associated distal DVT, further clinical research investigating the optimal doses of DOACs would be warranted.

The strength of the current study was a pre-specified subgroup analysis of the ONCO DVT study, which confirmed the consistent results with the primary report. The criterion for a reduced dose of edoxaban was determined based on the study protocol, which did not show an arbitrary selection bias by the physicians. In addition, the results of the per-protocol and as-treated analyses were also generally consistent with the primary results, which reinforced the robustness of the current study.

### Limitations

The current study had several limitations. First, the open-label design had the potential to introduce bias, such as ascertainment bias. However, all clinical endpoints were adjudicated by the members of the independent committee who were blinded to the study group assignments. The criterion for a reduced dose of edoxaban was determined based on the study protocol. However, there were a few patients in which the protocol deviated, and a potential selection bias could not be completely denied. Second, patients receiving a reduced dose of edoxaban were more common than those receiving a standard dose of edoxaban. A reduced dose of edoxaban for active cancer patients with VTE is reported to be more common in Asian populations,^20^ while the proportion of those patients receiving a reduced dose of edoxaban is reported to be low in Western populations.^16,21^ In addition, there could be racial differences including in the bleeding risk.^18^ Thus, the generalizability of the current results should be considered carefully including the application of the current results to non-Japanese populations.^22^ Third, we did not evaluate the smoking status, which is considered to be an important factor in the field of cardiovascular diseases, and the effect of that was unknown.

## Conclusions

A 12-month edoxaban regimen for cancer-associated isolated distal DVT was consistently superior to a 3-month regimen across the doses of edoxaban for the thrombotic risk, which suggested caution should be taken with the standard dose of edoxaban due to the potential for increasing the risk of bleeding with prolonged anticoagulation therapy.

## Supplementary Material

pvae028_Supplemental_File

## Notes

**Conflict of interest:** Y.Y. received lecture fees from Bayer Healthcare, Bristol-Myers Squibb, Pfizer, and Daiichi Sankyo, and grant support from Bayer Healthcare and Daiichi Sankyo. T.M. reports lecturer fees from AstraZeneca, Bristol-Myers Squibb, Daiichi Sankyo, Japan Lifeline, Kowa, Pfizer, and Tsumura; manuscript fees from Bristol-Myers Squibb and Pfizer; and advisory board for Novartis and Teijin. Y.N. received lecture fees from Bayer Healthcare, Bristol-Myers Squibb, Pfizer, and Daiichi Sankyo. Y.O. received lecture fees from Bayer Healthcare, Bristol-Myers Squibb, Pfizer, and Daiichi Sankyo, and research funds from Bayer Healthcare and Daiichi Sankyo. Dr Dohi received lecture fees from Bayer Healthcare, Bristol-Myers Squibb, Pfizer, and Daiichi Sankyo, and research funds from Daiichi Sankyo. N.I. received lecture fees from Bayer Healthcare, Bristol-Myers Squibb, and Daiichi Sankyo. Y.T. received lecture fees from AstraZeneca, Chugai Pharmaceutical, Bristol-Myers Squibb, Kyowa Kirin, Pfizer, Taiho Pharmaceutical, Takeda Pharmaceutical, and Daiichi Sankyo, and grant support from Daiichi Sankyo, AstraZeneca, and Ono Pharmaceutical. S.I. received lecture fees from Bayer Healthcare, Bristol-Myers Squibb, and Daiichi Sankyo. Dr Mo received lecture fees from Bayer Healthcare. All other authors have reported that they have no relationships relevant to the contents of this paper to disclose.

## Data availability

The data, analytic methods, and study materials will not be made available to other researchers for purposes of reproducing the results or replicating the procedure.
