# Supplementary material for: Edoxaban for 12 vs. 3 months in cancer-associated isolated distal deep vein thrombosis according to different doses: insights from the ONCO DVT study
Source: Eur Heart J Cardiovasc Pharmacother. 2024 Apr 22;10(5):422–31. doi: 10.1093/ehjcvp/pvae028 (PMC11323369; doi:10.1093/ehjcvp/pvae028)
Supplement: pvae028_Supplemental_File [file pvae028_supplemental_file.docx]

**Supplementary Material**

**Table of Contents**

[**Supplementary Appendix 2**](#_Toc156826695)

[Supplementary Appendix 1: Study Organization 2](#_Toc156826696)

[Supplementary Appendix 2: Participating Centers 5](#_Toc156826697)

[Supplementary Appendix 3: Definition of the baseline characteristics 8](#_Toc156826698)

[Supplementary Appendix 4: Definition of the Endpoints 9](#_Toc156826699)

[Supplementary Appendix 5: Analysis populations 10](#_Toc156826700)

[**Supplementary Tables 11**](#_Toc156826701)

[Table S1: Clinical characteristics of the patients at baseline 11](#_Toc156826702)

[Table S2: Types of cancer 13](#_Toc156826703)

[Table S3: Reasons for persistent edoxaban discontinuation 14](#_Toc156826704)

[Table S4: Per-protocol analysis for clinical outcomes 15](#_Toc156826705)

[Table S5: As-treated analysis for clinical outcomes 17](#_Toc156826706)

[Table S6: Detail of clinical events* 19](#_Toc156826707)

[Table S7: Subgroup analyses for the primary endpoint, major secondary endpoint, and all clinically relevant bleeding in the reduced dose (30 mg/day) edoxaban subgroup 21](#_Toc156826707)

[**Supplementary Figures 2**](#_Toc156826708)**4**

Figure S1: Kaplan–Meier curves for persistent edoxaban discontinuation comparing 12-month and 3-month edoxaban treatment groups in the subgroups stratified by the doses of edoxaban; (A) edoxaban 60 mg subgroup and (B) edoxaban 30 mg subgroup 23

[Figure S2: Study flow chart of per-protocol analysis 2](#_Toc156826709)4

[Figure S3: Per-protocol analysis for the primary endpoint 2](#_Toc156826710)5

[Figure S4: Study flow chart of an as-treated analysis 2](#_Toc156826711)6

[Figure S5: As-treated analysis for the primary endpoint 2](#_Toc156826712)7

[Figure S6: Kaplan–Meier curves for the asymptomatic recurrent VTE 28](#_Toc156826713)

[Figure S7: Kaplan–Meier curves for the all clinically relevant bleeding 2](#_Toc156826714)9

[Figure S8: Kaplan–Meier curves for all cause death 30](#_Toc156826715)

[Figure S9: Kaplan–Meier curves for major bleeding on edoxaban treatment 31](#_Toc156826716)

[Figure S10: Kaplan–Meier curves for the all clinically relevant bleeding on edoxaban treatment 32](#_Toc156826717)

# **Supplementary Appendix**

## **Supplementary Appendix 1: Study Organization**

**Principal Investigator:** Yugo YAMASHITA, Department of Cardiovascular Medicine, Graduate School of Medicine and Faculty of Medicine Kyoto University

**Protocol Committee:** Takeshi KIMURA (Co-Principal Investigators), Department of Cardiovascular Medicine, Graduate School of Medicine and Faculty of Medicine Kyoto University; Makoto MO, Department of Cardiovascular Surgery, Yokohama Minami Kyousai Hospital; Hisashi GOTO, Department of General Surgery/Department of Cardiovascular Medicine, Tohoku University; Daisuke SUETA, Department of Cardiovascular Medicine, Kumamoto University; Yugo YAMASHITA, Department of Cardiovascular Medicine, Graduate School of Medicine and Faculty of Medicine Kyoto University

**Steering Committee:** Norikazu YAMADA, Department of Cardiovascular Medicine, Kuwana City Medical Center; Taro SHIGA, Department of Cardiovascular Medicine, Cancer Institute Hospital; Tsuyoshi YAMAMOTO, Department of Cardiovascular Intensive Care, Nippon Medical School Hospital; Masafumi FUJITA, Department of Onco-Cardiology, Osaka International Cancer Institute; Kazunori OTSUI, Department of General Internal Medicine, Kobe University Hospital; Kenichi TSUJITA, Department of Cardiovascular Medicine, Kumamoto University; Satoshi IKEDA, Department of Cardiovascular Medicine, Nagasaki University; Norimichi KOITABASHI, Department of Cardiovascular Medicine, Gunma University; Shinji HISATAKE, Department of Cardiovascular Medicine, Toho University; Naohiko NAKANISHI, Department of Cardiovascular Medicine, Kyoto Prefectural University of Medicine; Kentaro JUJO, Department of Cardiovascular Medicine, Tokyo Women’s Medical University; Koichiro SUGIMURA, Department of General Surgery/Department of Cardiovascular Medicine, Tohoku University; Ryoji TAKEDA, Department of Vascular Surgery, Rakuwakai Otowa Hospital; Reo HATA, Department of Cardiovascular Medicine, Kurashiki Central Hospital; Kazushige KADOTA, Department of Cardiovascular Medicine, Kurashiki Central Hospital; Toru TAKASE, Department of Cardiovascular Medicine, Kindai University Hospital; Shunichi MIYAZAKI, Department of Cardiovascular Medicine, Kindai University Hospital; Seiichi HIRAMORI, Department of Cardiovascular Medicine, Kokura Memorial Hospital; Kenji ANDO, Department of Cardiovascular Medicine, Kokura Memorial Hospital; Kite KIM, Department of Cardiovascular Medicine, Kobe City Medical Center General Hospital; Yutaka FURUKAWA, Department of Cardiovascular Medicine, Kobe City Medical Center General Hospital; Jiro SAKAMOTO, Department of Cardiovascular Medicine, Tenri Hospital; Masaharu AKAO, Department of Cardiovascular Medicine, National Hospital Organization (NHO) Kyoto Medical Center

**Research Operations Staff:** Yusuke YOSHIKAWA, Department of Cardiovascular Medicine, Graduate School of Medicine and Faculty of Medicine Kyoto University

**Clinical Events Committee:** Yasuhiro HAMATANI, Department of Cardiovascular Medicine, NHO Kyoto Medical Center; Kensuke TAKABAYASHI, Department of Cardiovascular Medicine, Hirakata Kohsai Hospital; Yuji NISHIMOTO, Department of Cardiovascular Medicine, Hyogo Prefectural Amagasaki General Medical Center; Yukiko NAKANO, Department of Cardiovascular Medicine, Graduate School of Medicine and Faculty of Medicine Kyoto University

**Data Safety Monitoring Committee:** Mitsuru ABE Department of Cardiovascular Medicine, NHO Kyoto Medical Center; Hidenori YAKU, Department of Cardiovascular Medicine, Mitsubishi Kyoto Hospital

**Clinical Research Organization:** MID, Inc. and Department of Cardiovascular Medicine, Graduate School of Medicine and Faculty of Medicine Kyoto University

**Monitoring officers:** Yasuaki TAKEJI, Department of Cardiovascular Medicine, Kyoto University Hospital; Yusuke YOSHIKAWA, Department of Cardiovascular Medicine, Kyoto University Hospital

**Auditors:** Chikashi TAKEDA, Department of Pharmacoepidemiology, Kyoto University Graduate School of Medicine and Public Health; Aki KUWAUCHI, Department of Pharmacoepidemiology, Kyoto University Graduate School of Medicine and Public Health

**Principal Statistician:** Takeshi MORIMOTO, Department of Clinical Epidemiology, Hyogo College of Medicine

## **Supplementary Appendix 2: Participating Centers**

Department of Cardiovascular Medicine, Kyoto University Hospital (Yugo YAMASHITA), Department of Onco-Cardiology, Osaka International Cancer Institute (Masafumi FUJITA), Department of Cardiovascular Medicine, Saiseikai Noe Hospital (Ichiro KOUCHI), Department of Cardiology, Osaka Red Cross Hospital (Tsukasa INADA), Department of Cardiovascular Medicine, Japanese Red Cross Otsu Hospital (Kazuaki KAITANI), Department of Cardiovascular Medicine, Kakogawa Central City Clinics (Hiroaki NAKAMURA), Department of Cardiovascular Medicine, Cancer Institute Hospital (Taro SHIGA), Department of Vascular Surgery, Kansai Medical University Medical Center (Nobuko YAMAMOTO), Department of Cardiovascular Medicine, University Hospital Kyoto Prefectural University of Medicine (Satoaki MATOBA), Department of Cardiovascular Surgery, Kyorin University Faculty of Medicine (Yutaka HOSOI), Department of Cardiovascular Medicine, Kindai University Hospital (Gaku NAKAZAWA), Department of Cardiovascular Medicine, Kumamoto University Hospital (Daisuke SUETA), Department of Cardiovascular Medicine, Kurashiki Central Hospital (Kazushige KADOTA), Department of Cardiovascular Surgery, Kurume University Hospital (Shinichi HIROMATSU), Department of Cardiovascular Medicine, Kuwana City Medical Center (Norikazu YAMADA), Department of Cardiovascular Medicine, Gunma University (Norimichi KOITABASHI), Department of Cardiovascular Medicine, Kobe City Medical Center General Hospital (Yutaka FURUKAWA), Department of General Internal Medicine, Kobe University Hospital (Kazunori OTSUI), Department of Cardiovascular Medicine, Kohka Public Hospital (Tomohiro DOUKA), Department of Cardiovascular Surgery, Fukushima Medical University Hospital (Daiki WAKAMATSU), Department of Cardiovascular Medicine, Kokura Memorial Hospital (Kenji ANDO), Department of General Internal Medicine / Department of Cardiovascular Medicine, National Cancer Center Hospital (Masaaki SHOJI), Department of Cardiovascular Medicine, NHO Okayama Medical Center (Hiroto SHIMOKAWAHARA), Department of Cardiovascular Medicine, NHO Kyoto Medical Center (Kosuke DOI), Department of Cardiovascular Medicine, Saiseikai Yokohamashi Nanbu Hospital (Tsutomu ENDO), Department of Cardiovascular Surgery, Saiseikai Wakayama Hospital (Atsutoshi HATADA), Department of Cardiovascular Medicine, Saku Central Hospital Advanced Care Center (Yoshikazu YAZAKI), Department of Cardiovascular Medicine, Shiga General Hospital (Takeshi UENO), Department of Cardiovascular Medicine, Shizuoka Cancer Center (Nao MURAOKA), Department of Cardiovascular Medicine, Shizuoka City Shizuoka Hospital (Ryuzo NAWATA), Department of Respiratory Medicine and Clinical Oncology, Shimane University Hospital (Yukari TSUBATA), Department of Cardiovascular Medicine, Shimada General Medical Center (Yoshiaki TSUYUKI), Department of Cardiology, St. Marianna University School of Medicine (Yasuhiro TANABE), Department of Cardiovascular Medicine, Medical Research Institute Kitano Hospital (Moriaki INOKO), Department of Obstetrics and Gynecology, University of Tsukuba Hospital (Toyomi SATO), Department of Cardiovascular Medicine, Tenri Hospital (Toshihiro TAMURA), Department of Cardiovascular Medicine, Tokyo Women’s Medical University Hospital (Yuichiro MINAMI), Department of Cardiovascular Medicine, Tokyo Metropolitan Tama Medical Center (Hiroyuki TANAKA), Department of Cardiovascular Medicine, Toho University Ohashi Medical Center (Nobutaka IKEDA), Department of Cardiovascular Medicine, Toho University Omori Medical Center (Shinji HISATAKE), Department of General Surgery, Tohoku University Hospital (Hisashi GOTO), Department of Cardiovascular Medicine, Nagasaki University Hospital (Koji MAEMURA), Department of Obstetrics and Gynecology, Nara Medical University Hospital (Ryuji KAWAGUCHI), Department of Cardiovascular Intensive Care, Nippon Medical School Hospital (Tsuyoshi YAMAMOTO), Department of Cardiovascular Medicine, Japanese Red Cross Wakayama Medical Center (Shojiro TATSUSHIMA), Department of Cardiovascular Medicine, Hyogo Prefectural Amagasaki General Medical Center (Yukihiro SATO), Department of Cardiovascular Medicine, Hirakata Kohsai Hospital (Shoji KITAGUCHI), Department of Cardiovascular Medicine, Fukui Prefectural Hospital (Susumu FUJINO), Department of Vascular Surgery, Saiseikai Yahata General Hospital (Shinsuke MII), Department of Cardiovascular Medicine, Fujisawa City Hospital (Kengo TSUKAHARA), Department of Cardiovascular Medicine, Makiminato Central Hospital (Naoya MAEHIRA), Department of Cardiovascular Medicine, Mie University Hospital (Kaoru DOHI), Department of Cardiovascular Medicine, Mitsubishi Kyoto Hospital (Takafumi YOKOMATSU), Department of Cardiovascular Medicine, Japanese Red Cross Musashino Hospital (Takashi ASHIKAGA), Department of Cardiovascular Surgery, Yokohama Minami Kyousai Hospital (Makoto MO), Hospital Department of Cardiovascular Medicine, Yokohama Rosai Hospital (Kazuhiko YUMOTO), Department of Vascular Surgery, Rakuwakai Otowa Hospital (Ryoji TAKEDA), Department of Cardiovascular Medicine, Niigata University Graduate School of Medicine and Dentistry (Shinya FUJIKI), Department of Internal Medicine, Niigata Cancer Center Niigata Hospital (Yuji OKURA), Department of Surgery of the Lower Gastrointestinal Surgery, Hyogo College of Medicine (Jihyung SONG)

## **Supplementary Appendix 3: Definition of the baseline characteristics**

Diabetes: Blood glucose level ≥200 mg/dl 2 or more hours after loading in a glucose tolerance test, casual blood glucose ≥200 mg/dl, fasting blood glucose ≥126 mg/dl, or Hemoglobin A1c ≥6.5%. Even when the above tests are not performed diabetes is defined if the patient has already been clinically diagnosed with diabetes or is taking medication to treat diabetes. Heart failure: Cases that satisfy any of the following criteria are defined as heart failure: history of a hospitalization for heart failure, clinical heart failure symptoms at a New York Heart Association II (can walk on flat ground but cannot jog) or higher, or left ventricular ejection fraction <40%. History of major bleeding: Cases that satisfy any of the following criteria are defined as having a history of major bleeding. A history of bleeding into vital organs, history of bleeding that required a blood transfusion, history of bleeding with a reduction in the hemoglobin of ≥2 g/dl, or a history of bleeding that required fluid transfusion, vasopressors, or surgical treatment. Transient risk factors for venous thromboembolism included recent surgery, recent immobilization, long-distance travel, central venous catheter use, pregnancy or puerperium, recent leg trauma, fracture or burn, severe infection, and estrogen use. Anemia was diagnosed if the value of hemoglobin was <13 g/dL for men and <12 g/dL for women. Eastern Cooperative Oncology Group (ECOG) performance status (PS): 0, Fully active, at pre-disease performance levels without restriction. 1. Restricted physically strenuous activity, but ambulatory and able to carry out work of a light and sedentary nature. 2. Ambulatory and capable of all self-care but unable to carry out any work activities. Up and about more than 50% of waking hours. 3. Capable of only limited self-care, confined to bed or chair more than 50% of waking hours. 4. Completely disabled. Cannot carry on any self-care. Totally confined to bed or a chair. Transient risk factors for VTE included recent surgery, recent immobilization, long-distance travel, central venous catheter use, pregnancy or puerperium, recent leg trauma, fracture or burn, severe infection, and estrogen use.

## **Supplementary Appendix 4:** **Definition of the Endpoints**

- **Death**

Cause of death was classified into the following 5 categories. VTE-related deaths, death due to cancer, cardiovascular death, deaths due to bleeding, and deaths due to other known causes.

- VTE-related death: Death due to PE diagnosed prior to death or at autopsy, or death unexplained by other than PE
- Cancer: Deaths conceivably directly associated with cancer (example: brain herniation, coma, brain tumor causing respiratory arrest), and debilitating (gradual) death due to the progression of cancer
- Cardiovascular death: All cardiac and vascular deaths (myocardial infarction, low-output heart failure, lethal arrhythmia, cerebrovascular disease, ruptured aortic aneurysm, dissecting aneurysm, etc.)
- Bleeding: Deaths directly caused by hemorrhage (example: cerebral hemorrhage causing brain herniation, gastrointestinal hemorrhage resulting in death due to hemorrhagic shock, etc.)
- Other known causes: Deaths due to known causes not listed above. These include infection, renal failure, lung disease, intraoperative death, accident, suicide, trauma, etc.
- **Bleeding/Hemorrhagic Events**

The following severe major bleeding events will be evaluated by an independent Data and Safety Monitoring Committee.

- Category 1: Bleeding determined as clinically non-urgent
- Category 2: Less urgent bleeding that requires some clinical intervention but is not classified in other categories
- Category 3: Urgent bleeding, such as hemodynamic instability and intracranial bleeding
- Category 4: Bleeding directly linked to death, or that may lead to death shortly after admission

## **Supplementary Appendix 5: Analysis populations**

<Full analysis set>

All patients randomly assigned to the treatment group will be included, inclusive of all data obtained from first day of the group assignment until the end of the follow-up period.

<Per-protocol analysis>

Patients who were randomly assigned to and administered the study drug (edoxaban) at least once, with no major deviations from the research protocol, will be included, and data from the day of assignment to the end the follow-up period will be included. We defined 3-month edoxaban group as patients who did not receive edoxaban at 120 days after diagnosis who assigned to 3-month edoxaban group, and 12-month edoxaban group as patients who received edoxaban at 120 days after diagnosis who assigned to 12-month edoxaban group. We excluded patients enrolled with exclusion criteria at randomization, patients lost to follow-up before 120 days after diagnosis, and patients who died before 120 days after diagnosis.

<As-treated analysis>

Patients who were randomly assigned to and administered the study drug (edoxaban) at least once will be included, inclusive of all data obtained from the day of assignment until the end of the follow-up period. Regardless of randomly assigned group, 1) the patients who did not receive edoxaban at 120 days after diagnosis were defined as short edoxaban group, and 2) the patients who received edoxaban at 120 days after diagnosis were defined as long edoxaban group. We excluded patients enrolled with exclusion criteria at randomization, patients lost to follow-up before 120 days after diagnosis, and patients who died before 120 days after diagnosis.

# **Supplementary Tables**

## **Table S1: Clinical characteristics of the patients at baseline comparing 12-month and 3-month edoxaban group in standard dose (60 mg/day) and reduced dose (30 mg/day) of edoxaban subgroups**

|  | **Standard dose of edoxaban**  **(60 mg/day)**  **(N=151)** | | **Reduced dose of edoxaban**  **(30 mg/day)**  **(N=450)** | |
| --- | --- | --- | --- | --- |
|  | **12-month edoxaban**  **(N=80)** | **3-month edoxaban**  **(N=71)** | **12-month edoxaban**  **(N=216)** | **3-month edoxaban**  **(N=234)** |
| **Baseline characteristics** |  |  |  |  |
| Age, years | 67.4±10.0 | 67.8±9.7 | 70.7±10.4 | 70.7±10.4 |
| Age ≥75 years, No. (%) | 23 (29) | 20 (28) | 94 (40) | 94 (40) |
| Men, No. (%) | 46 (58) | 35 (49) | 38 (84) | 38 (84) |
| Body weight, kg | 71.2±9.3 | 69.4±8.1 | 50.4±8.5 | 50.4±8.5 |
| Body weight <60 kg, No. (%) | 2 (2.5) | 2 (2.8) | 220 (94) | 220 (94) |
| Body mass index, kg/m^2^ | 26.7±4.0 | 26.6±4.2 | 21.1±3.1 | 21.1±3.1 |
| Symptoms at baseline, No. (%) | 17 (21) | 10 (14) | 59 (25) | 59 (25) |
| Site of thrombosis, No. (%) |  |  |  |  |
| Bilateral, No. (%) | 30 (38) | 21 (30) | 84 (36) | 84 (36) |
| Right side, No. (%) | 24 (30) | 26 (37) | 55 (24) | 55 (24) |
| Left side, No. (%) | 26 (33) | 24 (34) | 95 (41) | 95 (41) |
| **Cancer status** |  |  |  |  |
| Newly diagnosed with cancer within 6 mo, No. (%) | 53 (66) | 50 (70) | 131 (61) | 155 (66) |
| Chemotherapy performed within 6 mo, No. (%) | 34 (43) | 34 (47) | 108 (50) | 107 (46) |
| Radiotherapy performed within 6 mo, No. (%) | 2 (2.5) | 6 (8.5) | 18 (8.3) | 26 (11) |
| Scheduled to be operated within 6 mo, No. (%) | 34 (43) | 27 (38) | 101 (47) | 94 (40) |
| Local invasion, No. (%) | 14 (18) | 12 (17) | 43 (20) | 51 (22) |
| Recurrent cancer, No. (%) | 4 (5.0) | 7 (9.9) | 27 (12) | 27 (12) |
| Metastatic disease, No. (%) | 15 (19) | 19 (27) | 61 (26) | 61 (26) |
| ECOG performance status, No. (%) |  |  |  |  |
| 0 | 48 (60) | 40 (56) | 110 (47) | 110 (47) |
| 1 | 21 (26) | 22 (31) | 81 (35) | 81 (35) |
| ≥2 | 11 (14) | 9 (13) | 43 (18) | 43 (18) |
| **Comorbidities** |  |  |  |  |
| Hypertension, No. (%) | 42 (53) | 35 (49) | 95 (51) | 95 (51) |
| Diabetes, No. (%) | 27 (34) | 19 (27) | 28 (12) | 28 (12) |
| Heart failure, No. (%) | 4 (5.0) | 0 | 3 (1.3) | 3 (1.3) |
| History of stroke, No. (%) | 5 (6.2) | 3 (4.2) | 10 (4.3) | 10 (4.3) |
| History of VTE, No. (%) | 6 (7.5) | 4 (5.6) | 9 (3.8) | 9 (3.8) |
| History of major bleeding, No. (%) | 2 (2.5) | 5 (7.0) | 11 (4.7) | 11 (4.7) |
| Transient risk factors for VTE, No. (%) | 21 (26) | 16 (23) | 55 (24) | 55 (24) |
| Recent surgery within 2 months, No. (%) | 9 (11) | 13 (18) | 31 (13) | 31 (13) |
| **Laboratory tests at diagnosis** |  |  |  |  |
| Creatinine clearance, mL/min | 86.1 (69.2-105.8) | 86.5 (72.2-107.9) | 56.4 (46.9-73.6) | 61.9 (48.2-77.4) |
| Creatinine clearance ≤50 mL/min, No. (%) | 2 (2.5) | 1 (1.4) | 67 (31) | 61 (26) |
| Anemia, No. (%) | 43 (54) | 44 (62) | 159 (68) | 159 (68) |
| Platelet count <100,000 per μl, No. (%) | 3 (3.8) | 6 (8.5) | 13 (5.6) | 13 (5.6) |
| D-dimer, μg/mL‖ | 5.0 (2.0-10.0) | 4.0 (2.0-10.0) | 5.0 (2.0-12.0) | 5.0 (2.0-12.0) |
| **Concomitant medication** |  |  |  |  |
| Antiplatelet, No. (%) | 7 (8.8) | 3 (4.2) | 18 (7.7) | 18 (7.7) |
| Steroid, No. (%) | 4 (2.6) | 9 (12.7) | 34 (15) | 34 (15) |
| Statins, No. (%) | 19 (24) | 16 (23) | 47 (20) | 47 (20) |

Values are expressed as the mean ± standard deviation, median (interquartile range], or number with percentage.

In the study protocol, edoxaban was recommended to be administered at a reduced dose of 30 mg once daily in patients with a creatinine clearance of 30 to 50 mL per minute or a body weight of 60 kg or less or in those receiving concomitant treatment with potent P-glycoprotein inhibitors. ECOG performance status values range from 0 to 4, with higher values indicating greater disability. Transient risk factors for VTE included recent surgery, recent immobilization, long-distance travel, central venous catheter use, pregnancy or puerperium, recent leg trauma, fracture or burn, severe infection, and estrogen use. Anemia was diagnosed if the value of hemoglobin was <13 g/dL for men and <12 g/dL for women. Values of D-dimer were missing in 34 patients.

Abbreviations: ECOG, Eastern Cooperative Oncology Group; VTE, venous thromboembolism

## **Table S2: Types of cancer**

| **Types of cancer, No. (%)** | **Standard dose of edoxaban**  **(60 mg/day)**  **(N=151)** | | **Reduced dose of edoxaban**  **(30 mg/day)**  **(N=450)** | |
| --- | --- | --- | --- | --- |
|  | **12-month edoxaban**  **(N=80)** | **3-month edoxaban**  **(N=71)** | **12-month edoxaban**  **(N=216)** | **3-month edoxaban**  **(N=234)** |
| Ovary | 10 (13) | 7 (9.9) | 32 (15) | 41 (18) |
| Uterus | 7 (8.8) | 13 (18) | 30 (14) | 31 (13) |
| Lung | 12 (15) | 5 (7.0) | 26 (12) | 29 (12) |
| Colon | 9 (11) | 3 (4.2) | 18 (8.3) | 28 (12) |
| Pancreas | 7 (8.8) | 6 (8.5) | 18 (8.3) | 22 (9.4) |
| Stomach | 6 (7.5) | 5 (7.0) | 9 (4.2) | 13 (5.6) |
| Blood | 4 (5.0) | 2 (2.8) | 11 (5.1) | 16 (6.8) |
| Breast | 4 (5.0) | 3 (4.2) | 17 (7.9) | 9 (3.8) |
| Bladder | 6 (7.5) | 3 (4.2) | 5 (2.3) | 9 (3.8) |
| Kidney/ureter | 0 (0) | 3 (4.2) | 6 (2.8) | 4 (1.7) |
| Skin | 1 (1.2) | 5 (7.0) | 3 (1.4) | 1 (0.4) |
| Prostate | 2 (2.5) | 2 (2.8) | 5 (2.3) | 3 (1.3) |
| Liver | 1 (1.2) | 1 (1.4) | 4 (1.9) | 4 (1.7) |
| Brain | 1 (1.2) | 2 (2.8) | 6 (2.8) | 2 (0.9) |
| Esophagus | 2 (2.5) | 3 (4.2) | 5 (2.3) | 1 (0.4) |
| Gall bladder/bile duct | 0 (0) | 0 (0) | 4 (1.9) | 2 (0.9) |
| Thyroid gland | 1 (1.2) | 0 (0) | 0 (0) | 0 (0) |
| Multiple | 1 (1.2) | 2 (2.8) | 6 (2.8) | 3 (1.3) |
| Others | 6 (7.5) | 6 (8.5) | 11 (5.1) | 16 (6.8) |

## **Table S3: Reasons for persistent edoxaban discontinuation**

| **Reasons, No. (%)** | **Standard dose of edoxaban**  **(60 mg/day)** | | **Reduced dose of edoxaban**  **(30 mg/day)** | |
| --- | --- | --- | --- | --- |
|  | **12-month edoxaban**  **(N=34)** | **3-month edoxaban**  **(N=64)** | **12-month edoxaban**  **(N=82)** | **3-month edoxaban**  **(N=213)** |
| Per-protocol discontinuation | 0 (0) | 50 (78) | 0 (0) | 166 (78) |
| Due to bleeding events | 15 (44) | 2 (3.1) | 15 (18) | 19 (8.9) |
| Due to drug side effect | 1 (2.9) | 1 (1.6) | 9 (11) | 4 (1.9) |
| Due to cancer progression | 6 (18) | 5 (7.8) | 23 (28) | 4 (1.9) |
| Due to operation | 2 (5.9) | 3 (4.7) | 8 (9.8) | 1 (0.5) |
| Patient’s decision | 3 (8.8) | 0 (0) | 11 (13) | 3 (1.4) |
| Physician’s decision | 3 (8.8) | 0 (0) | 6 (7.3) | 0 (0) |
| Others | 4 (12) | 3 (4.7) | 10 (12) | 16 (7.5) |

## **Table S4: Per-protocol analysis for clinical outcomes at 12 months in the standard dose (60 mg/day) and reduced dose (30 mg/day) edoxaban subgroups ***

|  | **N of patients with event** | | **Odds ratio (95% CI)** |
| --- | --- | --- | --- |
|  | **12-month edoxaban**  **(N=223)** | **3-month edoxaban**  **(N=234)** |  |
| **Primary endpoint** |  |  |  |
| 60 mg/day | 0/61 (0%) | 7/53 (13.2%) | - |
| 30 mg/day | 1/162 (0.6%) | 12/181 (6.6%) | 0.09 (0.01-0.68) |
| **Major secondary endpoint†** |  |  |  |
| 60 mg/day | 3/61 (4.9%) | 2/53 (3.8%) | 1.32 (0.21-8.21) |
| 30 mg/day | 9/162 (5.6%) | 10/181 (5.5%) | 1.01 (0.40-2.54) |
| **Other secondary endpoints** |  |  |  |
| **Symptomatic VTE recurrence events** |  |  |  |
| 60 mg/day | 0/61 (0%) | 7/53 (13.2%) | - |
| 30 mg/day | 1/162 (0.6%) | 12/181 (6.6%) | 0.09 (0.01-0.68) |
| **VTE-related deaths‡** |  |  |  |
| 60 mg/day | 0/61 (0%) | 0/53 (0%) | - |
| 30 mg/day | 0/162 (0%) | 0/181 (0%) | - |
| **Asymptomatic recurrent VTE§** |  |  |  |
| 60 mg/day | 8/61 (13.1%) | 9/53 (17.0%) | 0.74 (0.26-2.07) |
| 30 mg/day | 8/162 (4.9%) | 31/181 (17.1%) | 0.25 (0.11-0.57) |
| **All clinically relevant bleeding events¶** |  |  |  |
| 60 mg/day | 8/61 (13.1%) | 4/53 (7.5%) | 1.85 (0.52-6.53) |
| 30 mg/day | 18/162 (11.1%) | 25/181 (13.8%) | 0.78 (0.41-1.49) |
| **Deaths from all causes** |  |  |  |
| 60 mg/day | 4/61 (6.6%) | 8/53 (15.1%) | 0.40 (0.11-1.40) |
| 30 mg/day | 23/162 (14.2%) | 32/181 (17.7%) | 0.77 (0.43-1.38) |

*Patients who were randomly assigned to and administered the study drug (edoxaban) at least once, with no major deviations from the research protocol, were included, and data from the day of assignment to the end the follow-up period were included. We defined the 3-month edoxaban group as patients who did not receive edoxaban at 120 days after the diagnosis who were assigned to the 3-month edoxaban group, and 12-month edoxaban group as those patients who received edoxaban at 120 days after the diagnosis and who were assigned to the 12-month edoxaban group. We excluded patients enrolled with exclusion criteria at randomization, patients lost to follow-up before 120 days after diagnosis, and patients who died before 120 days after diagnosis. The 95% confidence intervals have not been adjusted for multiple comparisons.

†Major and nonmajor bleeding events were classified according to the criteria of the International Society on Thrombosis and Hemostasis.

‡Death due to pulmonary embolism diagnosed prior to death or at autopsy, or death unexplained by other than pulmonary embolism.

§Appearance of new or worsening thrombus images in the pulmonary arteries and deep veins on imaging tests (ultrasonography of lower limb vein system, computed tomography examination, pulmonary perfusion scintigraphy, pulmonary angiography, venography) that do not match the definition of symptomatic venous thromboembolism recurrence and are not associated with new or worsening symptoms.

¶For patients who had more than one event, only the first was counted.

## **Table S5: As-treated analysis for clinical outcomes at 12 months in the standard dose (60 mg/day) and reduced dose (30 mg/day) edoxaban subgroups***

|  | **N of patients with event** | | **Odds ratio (95% CI)** |
| --- | --- | --- | --- |
|  | **Long edoxaban (N=263)** | **Short edoxaban (N=279)** |  |
| **Primary endpoint** |  |  |  |
| 60 mg/day | 0/72 (0%) | 8/64 (12.5%) | - |
| 30 mg/day | 1/191 (0.5%) | 13/215 (6.0%) | 0.08 (0.01-0.63) |
| **Major secondary endpoint†** |  |  |  |
| 60 mg/day | 3/72 (4.2%) | 5/64 (7.8%) | 0.51 (0.12-2.24) |
| 30 mg/day | 13/191 (6.8%) | 16/215 (7.4%) | 0.91 (0.43-1.94) |
| **Other secondary endpoints** |  |  |  |
| **Symptomatic VTE recurrence events** |  |  |  |
| 60 mg/day | 0/72 (0%) | 8/64 (12.5%) | - |
| 30 mg/day | 1/191 (0.5%) | 13/215 (6.0%) | 0.08 (0.01-0.63) |
| **VTE-related deaths‡** |  |  |  |
| 60 mg/day | 0/72 (0%) | 0/64 (0%) | - |
| 30 mg/day | 0/191 (0%) | 0/215 (0%) | - |
| **Asymptomatic recurrent VTE§** |  |  |  |
| 60 mg/day | 9/72 (12.5%) | 12/64 (18.8%) | 0.62 (0.24-1.58) |
| 30 mg/day | 13/191 (6.8%) | 35/215 (16.3%) | 0.38 (0.19-0.73) |
| **All clinically relevant bleeding events¶** |  |  |  |
| 60 mg/day | 8/72 (11.1%) | 11/64 (17.2%) | 0.60 (1.23-1.61) |
| 30 mg/day | 23/191 (12.0%) | 34/215 (15.8%) | 0.73 (0.41-1.29) |
| **Deaths from all causes** |  |  |  |
| 60 mg/day | 5/72 (6.9%) | 11/64 (17.2%) | 0.36 (0.12-1.10) |
| 30 mg/day | 29/191 (15.2%) | 44/215 (20.5%) | 0.70 (0.42-1.17) |

*Patients who were randomly assigned to and administered the study drug (edoxaban) at least once were included, inclusive of all data obtained from the day of assignment until the end of the follow-up period. Regardless of randomly assigned group, 1) the patients who did not receive edoxaban at 120 days after the diagnosis were defined as the short edoxaban group, and 2) the patients who received edoxaban at 120 days after diagnosis were defined as the long edoxaban group. We excluded patients enrolled with exclusion criteria at randomization, patients lost to follow-up before 120 days after diagnosis, and patients who died before 120 days after the diagnosis. The 95% confidence intervals have not been adjusted for multiple comparisons.

†Major and nonmajor bleeding events were classified according to the criteria of the International Society on Thrombosis and Hemostasis.

‡Death due to pulmonary embolism diagnosed prior to death or at autopsy, or death unexplained by other than pulmonary embolism.

§Appearance of new or worsening thrombus images in the pulmonary arteries and deep veins on imaging tests (ultrasonography of lower limb vein system, computed tomography examination, pulmonary perfusion scintigraphy, pulmonary angiography, venography) that do not match the definition of symptomatic venous thromboembolism recurrence and are not associated with new or worsening symptoms.

¶For patients who had more than one event, only the first was counted.

## **Table S6: Detail of clinical events***

|  | **Standard dose of edoxaban**  **(60 mg/day)**  **(N=151)** | | **Reduced dose of edoxaban**  **(30 mg/day)**  **(N=450)** | |
| --- | --- | --- | --- | --- |
|  | **12-month edoxaban**  **(N=80)** | **3-month edoxaban**  **(N=71)** | **12-month edoxaban**  **(N=216)** | **3-month edoxaban**  **(N=234)** |
| **Symptomatic VTE recurrence events, No. (%)** | 1 (1.3) | 7 (9.9) | 2 (0.9) | 15 (6.4) |
| Types of VTE, No. (%) |  |  |  |  |
| Fatal PE | 0 (0) | 0 (0) | 0 (0) | 0 (0) |
| Nonfatal PE with or without DVT | 0 (0) | 1 (1.4) | 1 (0.5) | 1 (0.4) |
| PE with hypoxia | 0 (0) | 0 (0) | 1 (0.5) | 1 (0.4) |
| PE with shock | 0 (0) | 0 (0) | 0 (0) | 0 (0) |
| Deep vein thrombosis only | 1 (1.3) | 7 (9.9) | 1 (0.5) | 14 (6.0) |
| Proximal DVT | 0 (0) | 1 (1.4) | 1 (0.5) | 6 (2.6) |
| Distal deep vein thrombosis | 1 (1.3) | 6 (8.5) | 0 (0) | 7 (3.0) |
| Others | 0 (0) | 0 (0) | 0 (0) | 1 (0.4) |
| **Major bleeding, No. (%)†** | 11 (14) | 3 (4.2) | 17 (7.9) | 19 (8.1) |
| Before discontinuation of edoxaban, No. (% of patients with major bleeding) | 11/11 (100) | 1/3 (33) | 14/17 (83) | 15/19 (79) |
| Sites of major bleeding, No. (%) |  |  |  |  |
| Intracranial | 2 (2.5) | 0 (0) | 1 (0.5) | 1 (0.4) |
| Gastrointestinal (Upper) | 2 (2.5) | 0 (0) | 2 (0.9) | 6 (2.6) |
| Gastrointestinal (Lower) | 6 (7.5) | 2 (2.8) | 9 (4.2) | 7 (3.0) |
| Urogenital | 1 (1.3) | 0 (0) | 0 (0) | 3 (1.3) |
| Respiratory tract | 0 (0) | 0 (0) | 0 (0) | 1 (0.4) |
| Others | 0 (0) | 1 (1.4) | 5 (2.3) | 1 (0.4) |
| Severity of major bleeding, No. (% of patients with major bleeding)‡ |  |  |  |  |
| Category 1 | 0/11 (0) | 1/3 (33) | 0/17 (0) | 0/19 (0) |
| Category 2 | 8/11 (73) | 1/3 (33) | 16/17 (94) | 14/19 (74) |
| Category 3 | 2/11 (18) | 1/3 (33) | 1/17 (5.9) | 3/19 (16) |
| Category 4 | 1/11 (9.1) | 0/3 (0) | 0/17 (0) | 2/19 (11) |
| **All clinically relevant bleeding, No. (%)**§ | 22 (28) | 6 (8.5) | 31 (14) | 35 (15) |
| Before discontinuation of edoxaban, No. (% of patients with major bleeding) | 22/22 (100) | 3/6 (50) | 28/31 (90) | 26/35 (74) |
| Sites of all bleeding, No. (%) |  |  |  |  |
| Intracranial | 2 (2.5) | 0 (0) | 1 (0.5) | 1 (0.4) |
| Gastrointestinal (Upper) | 3 (3.8) | 0 (0) | 3 (1.4) | 5 (2.1) |
| Gastrointestinal (Lower) | 6 (7.5) | 2 (2.8) | 10 (4.6) | 11 (4.7) |
| Urogenital | 4 (5.0) | 1 (1.4) | 4 (1.9) | 5 (2.1) |
| Respiratory tract | 2 (2.5) | 0 (0) | 2 (0.9) | 1 (0.4) |
| Genital | 0 (0) | 0 (0) | 2 (0.9) | 3 (1.3) |
| Skin | 1 (1.3) | 0 (0) | 3 (1.4) | 5 (2.1) |
| Others | 4 (5.0) | 3 (4.2) | 6 (2.8) | 4 (1.7) |

*The analyses included all the patients who had undergone randomization after excluding patients who withdrew consent. For patients who did not experience an event, the time to first event was to be censored at day 365, or the last day the subject had a complete assessment for study outcomes, whichever comes first.

†Major bleeding events were classified according to the criteria of the International Society on Thrombosis and Hemostasis.

‡The severity of major bleeding at clinical presentation was adjudicated by an independent clinical events committee (whose members were unaware of the treatment assignments) according to the following prespecified categories: category 1 included bleeding events that were not considered to be a clinical emergency; category 2 included bleeding events that could not be classified in any of the other categories because they led to some treatment but were not considered to be a clinical emergency; category 3 included bleeding events that were considered to be a clinical emergency, such as bleeding with hemodynamic instability or intracranial bleeding with neurologic symptoms; and category 4 included bleeding events that led to death before or almost immediately after the patient entered the hospital.

§Clinically relevant bleeding events included major and non-major bleeding events. Clinically relevant non-major bleeding was defined as clinically overt bleeding (including bleeds detected only using imaging) not meeting the criteria for a major bleeding yet leading to one or more of the following: physician-guided medical intervention, hospital admission or further treatment for bleeding, or in person medical examination by a physician.

## **Table S7: Further subgroup analyses for the primary endpoint and major secondary endpoint among the reduced dose (30 mg/day) of edoxaban subgroup**

|  | **N of patients with event** | | **Odds ratio (95% CI)** |
| --- | --- | --- | --- |
|  | **12-month edoxaban (N=216)** | **3-month edoxaban (N=234)** |  |
| **Primary endpoint** |  |  |  |
| **Age** |  |  |  |
| ­≥75 years | 0/108 (0%) | 3/94 (3.2%) | - |
| <75 years | 2/108 (1.9%) | 12/140 (8.6%) | 0.20 (0.04-0.92) |
| **Body weight** |  |  |  |
| <60 kg | 2/197 (1.0%) | 14/220 (6.4%) | 0.15 (0.03-2.67) |
| ≥60 kg | 0/19 (0%) | 1/14 (7.1%) | - |
| **Creatinine clearance** |  |  |  |
| ≤50 mL/min | 1/67 (1.5%) | 5/61 (8.2%) | 0.17 (0.02-1.50) |
| >50 mL/min | 1/149 (0.7%) | 10/173 (5.8%) | 0.11 (0.01-0.87) |
| **Major secondary endpoint**† |  |  |  |
| **Age** |  |  |  |
| ≥75 years | 8/108 (7.4%) | 5/94 (5.3%) | 1.42 (0.45-4.51) |
| <75 years | 9/108 (8.3%) | 14/140 (10.0%) | 0.82 (0.34-1.97) |
| **Body weight** |  |  |  |
| <60 kg | 14/197 (7.1%) | 19/220 (8.6%) | 0.81 (0.39-1.66) |
| ≥60 kg | 3/19 (15.8%) | 0/14 (0%) | - |
| **Creatinine clearance** |  |  |  |
| ≤50 mL/min | 7/67 (10.4%) | 9/61 (14.8%) | 0.67 (0.24-1.94) |
| >50 mL/min | 10/149 (6.7%) | 10/173 (5.8%) | 1.17 (0.47-2.90) |

†Major bleeding events were classified according to the criteria of the International Society on Thrombosis and Hemostasis.

# **Supplementary Figures**

## **Figure S1: Kaplan–Meier curves for persistent edoxaban discontinuation comparing 12-month and 3-month edoxaban treatment groups in the subgroups stratified by the doses of edoxaban; (A) edoxaban 60 mg subgroup and (B) edoxaban 30 mg subgroup**


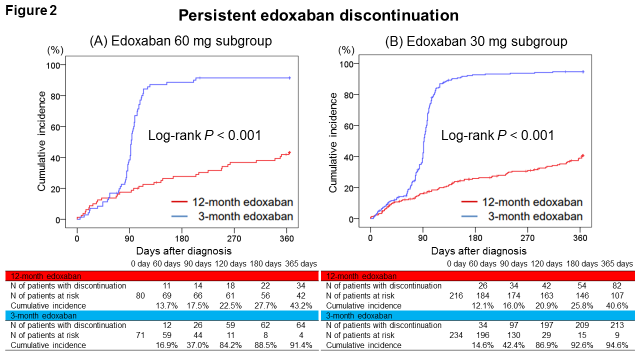


Time-to-event curves for persistent edoxaban discontinuation. Persistent edoxaban discontinuation was defined as the discontinuation of edoxaban according to the study protocol or lasting > 14 days for any reason.

## **Figure S2: Study flow chart of per-protocol analysis**


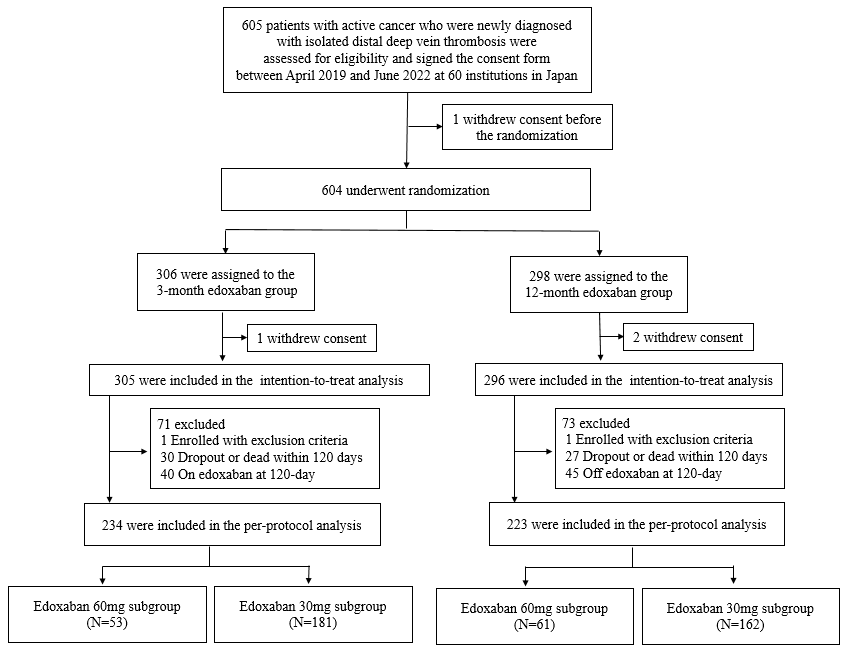


Patients who were randomly assigned to and administered the study drug (edoxaban) at least once, with no major deviations from the research protocol, were included, and data from the day of assignment to the end of the follow-up period were included. We defined the 3-month edoxaban group as those patients who did not receive edoxaban at 120 days after diagnosis who assigned to 3-month edoxaban group, and the 12-month edoxaban group as those patients who received edoxaban at 120 days after the diagnosis who were assigned to the 12-month edoxaban group. We excluded those patients enrolled with exclusion criteria at randomization, patients lost to follow-up before 120 days after diagnosis, and patients who died before 120 days after diagnosis.

## **Figure S3: Per-protocol analysis for the primary endpoint**

**(A) Edoxaban 60 mg subgroup, and (B) Edoxaban 30 mg subgroup**

**
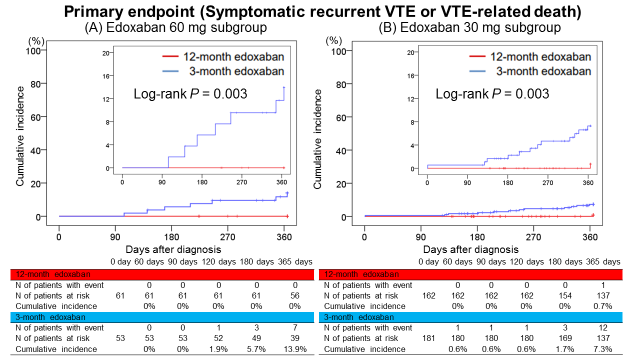
**

The time-to-event curves over 1-year after the diagnosis for the primary endpoint (symptomatic recurrent VTE or VTE-related death). Patients who were randomly assigned to and administered the study drug (edoxaban) at least once, with no major deviations from the research protocol, were included, and data from the day of assignment to the end of the follow-up period were included. We defined the 3-month edoxaban group as those patients who did not receive edoxaban at 120 days after the diagnosis who were assigned to the 3-month edoxaban group, and the 12-month edoxaban group as patients who received edoxaban at 120 days after the diagnosis who assigned to the 12-month edoxaban group. We excluded patients enrolled with exclusion criteria at randomization, patients lost to follow-up before 120 days after diagnosis, and patients who died before 120 days after the diagnosis.

VTE, venous thromboembolism.

## **Figure S4: Study flow chart of an as-treated analysis**


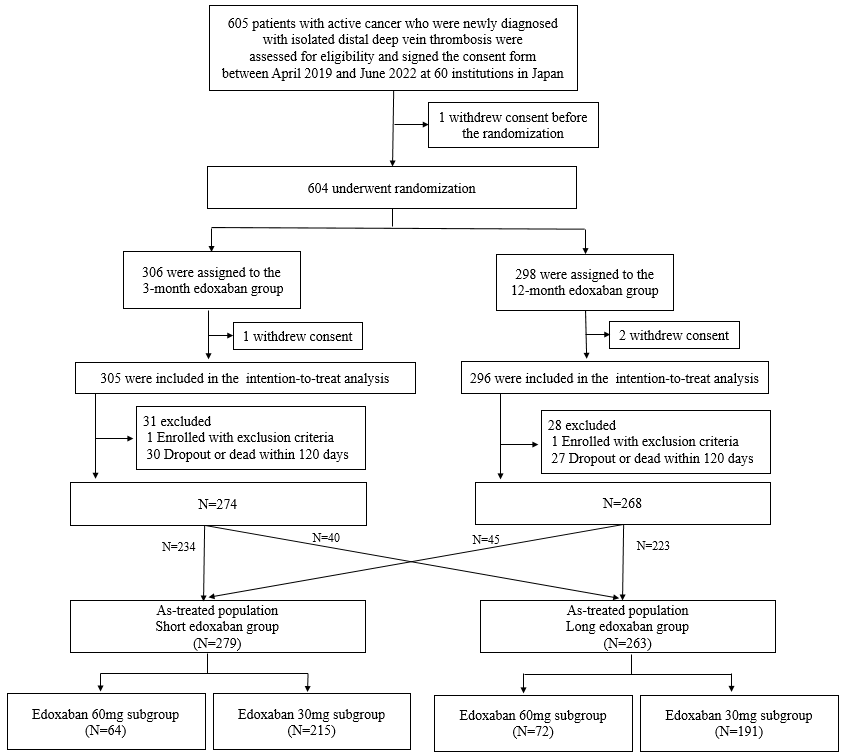


Patients who were randomly assigned to and administered the study drug (edoxaban) at least once were included, inclusive of all data obtained from the day of assignment until the end of the follow-up period. Regardless of randomly assigned group, 1) the patients who did not receive edoxaban at 120 days after diagnosis were defined as the short edoxaban group, and 2) the patients who received edoxaban at 120 days after diagnosis were defined as the long edoxaban group. We excluded patients enrolled with exclusion criteria at randomization, patients lost to follow-up before 120 days after the diagnosis, and patients who died before 120 days after the diagnosis.

VTE, venous thromboembolism.

## **Figure S5: As-treated analysis for the primary endpoint**

**(A) Edoxaban 60 mg subgroup, and (B) Edoxaban 30 mg subgroup**

**
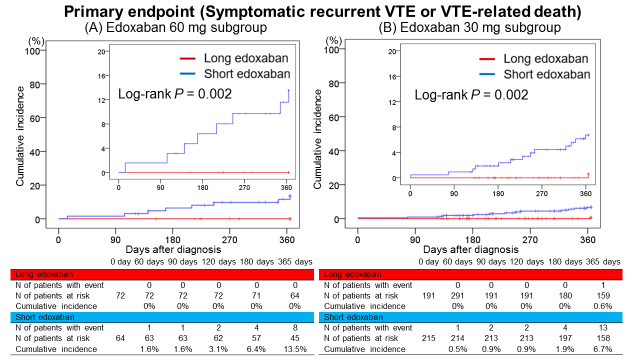
**

Time-to-event curves during 1-year after diagnosis for the primary endpoint (symptomatic recurrent VTE or VTE-related death). Patients who were randomly assigned to and administered the study drug (edoxaban) at least once were included, inclusive of all data obtained from the day of assignment until the end of the follow-up period. Regardless of the randomly assigned group, 1) the patients who received edoxaban at 120 days after diagnosis were defined as the long edoxaban group, and 2) the patients who did not receive edoxaban at 120 days after diagnosis were defined as the short edoxaban group. We excluded patients enrolled with exclusion criteria at randomization, patients lost to follow-up before 120 days after the diagnosis, and patients who died before 120 days after the diagnosis.

VTE, venous thromboembolism.

## **Figure S6: Kaplan–Meier curves for the asymptomatic recurrent VTE comparing the 12-month and 3-month edoxaban treatment groups in the subgroups stratified by the edoxaban dose**

**(A) Edoxaban 60 mg subgroup, and (B) Edoxaban 30 mg subgroup**


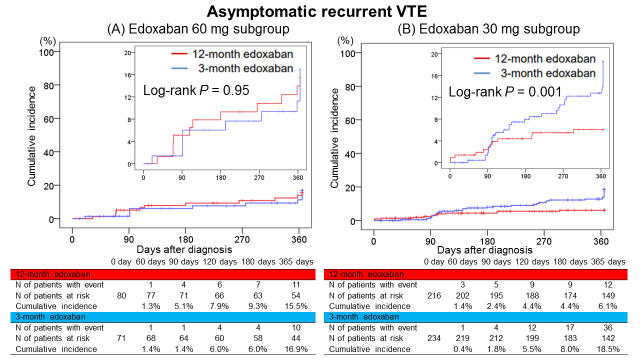


Time-to-event curves for the asymptomatic recurrent VTE 1 year after DVT diagnosis.

DVT, deep vein thrombosis; VTE, venous thromboembolism.

## **Figure S7: Kaplan–Meier curves for the all clinically relevant bleeding comparing the 12-month and 3-month edoxaban treatment groups in the subgroups stratified by the edoxaban dose**

**(A) Edoxaban 60 mg subgroup, and (B) Edoxaban 30 mg subgroup**


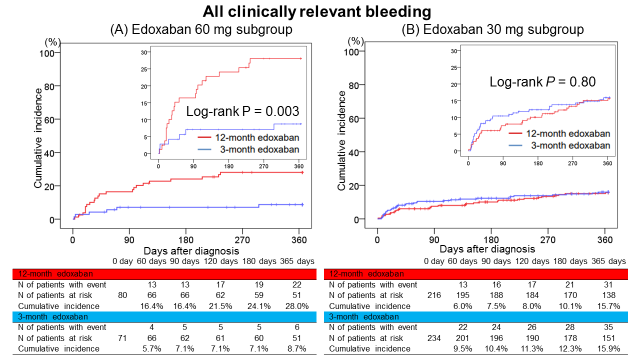


Time-to-event curves for all clinically relevant bleeding 1 year after DVT diagnosis.

Clinically relevant bleeding events included major and non-major bleeding events. Clinically relevant non-major bleeding was defined as clinically overt bleeding (including bleeds detected only using imaging) not meeting the criteria for a major bleeding yet leading to one or more of the following: physician-guided medical intervention, hospital admission or further treatment for bleeding, or in person medical examination by a physician.

DVT, deep vein thrombosis.

## **Figure S8: Kaplan–Meier curves for all cause death comparing the 12-month and 3-month edoxaban treatment groups in the subgroups stratified by the edoxaban dose**

**(A) Edoxaban 60 mg subgroup, and (B) Edoxaban 30 mg subgroup**


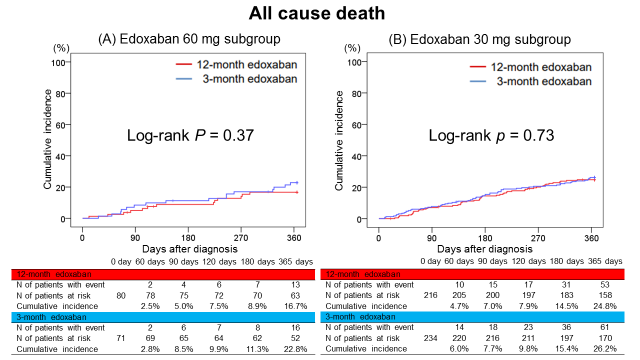


Time-to-event curves for all cause death 1 year after DVT diagnosis.

DVT, deep vein thrombosis.

## **Figure S9: Kaplan–Meier curves for major bleeding on edoxaban treatment comparing the 12-month and 3-month edoxaban treatment groups in the subgroups stratified by the edoxaban dose**

**(A) Edoxaban 60 mg subgroup, and (B) Edoxaban 30 mg subgroup**


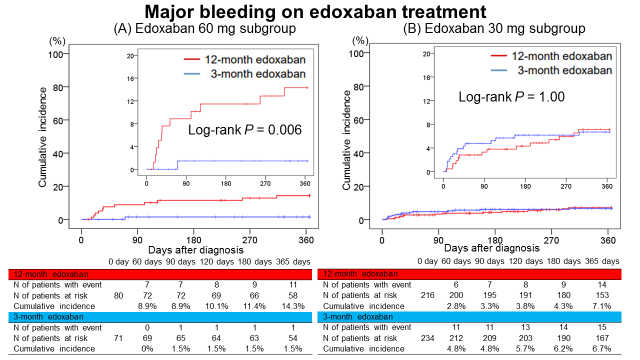


Time-to-event curves for the major bleeding on edoxaban treatment 1 year after DVT diagnosis.

Major bleeding was defined according to the International Society on Thrombosis and Haemostasis criteria, and comprised fatal bleeding, symptomatic bleeding in a critical area or organ, and bleeding causing a reduction in the hemoglobin levels ≥ 2 g/dL or leading to a transfusion of ≥ 2 units of whole blood or red cells.

## **Figure S10: Kaplan–Meier curves for the all clinically relevant bleeding on edoxaban treatment comparing the 12-month and 3-month edoxaban treatment groups in subgroups stratified by edoxaban dose**

**(A) Edoxaban 60 mg subgroup, and (B) Edoxaban 30 mg subgroup**


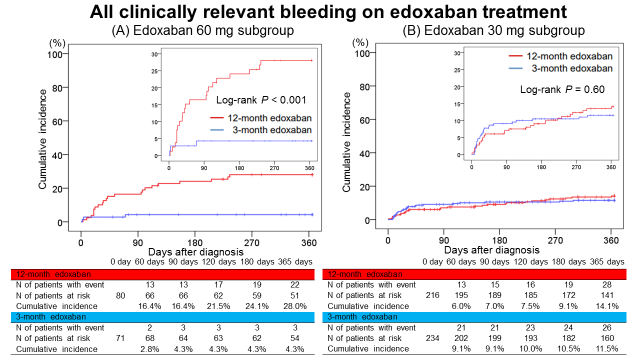


Time-to-event curves for all clinically relevant bleeding on edoxaban treatment 1 year after DVT diagnosis.

Clinically relevant bleeding events included major and non-major bleeding events. Clinically relevant non-major bleeding was defined as clinically overt bleeding (including bleeds detected only using imaging) not meeting the criteria for a major bleeding yet leading to one or more of the following: physician-guided medical intervention, hospital admission or further treatment for bleeding, or in person medical examination by a physician.

DVT, deep vein thrombosis.
